# Supplementary figures and images for: The Bee Gut Microbiota: Bridging Infective Agents Potential in the One Health Context
Source: Int J Mol Sci. 2024 Mar 27;25(7):3739. doi: 10.3390/ijms25073739 (PMC11012054; doi:10.3390/ijms25073739)

## Slide 1
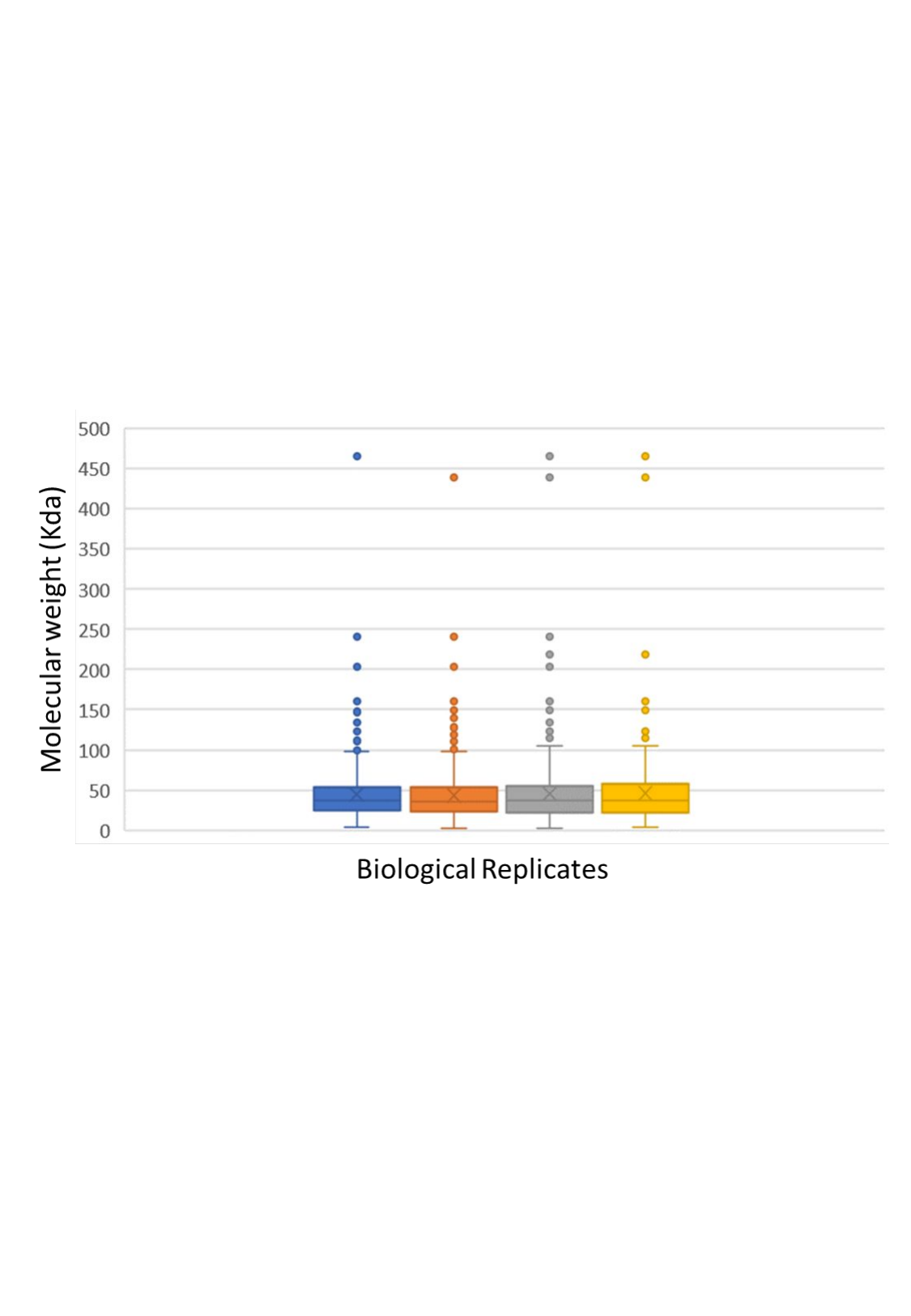

Supplement: Supplementary file 1 [file ijms-25-03739-s001.zip › Figure S1.pptx]

## Slide 1
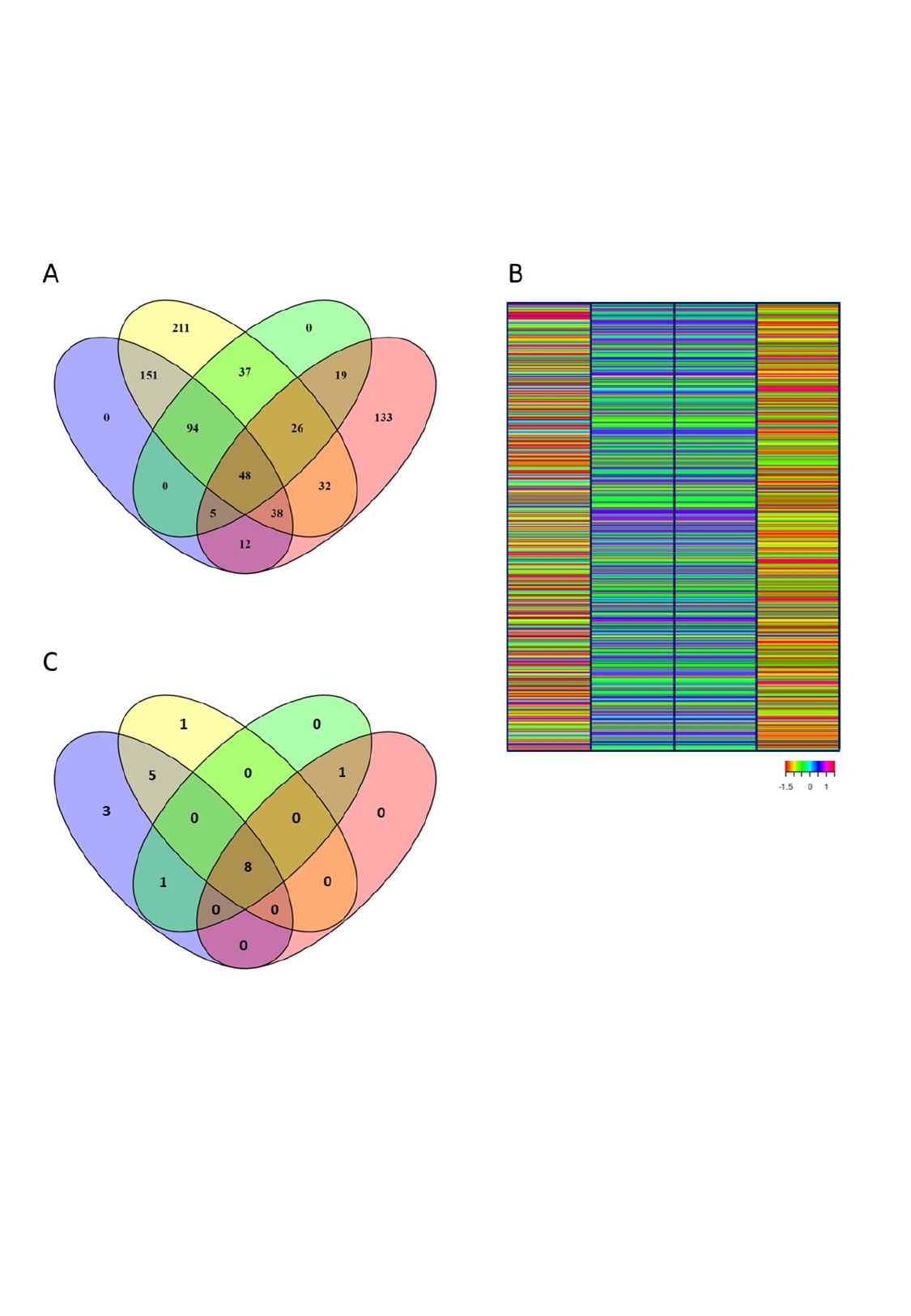

Supplement: Supplementary file 1 [file ijms-25-03739-s001.zip › Figure S2.pptx]

## Slide 1
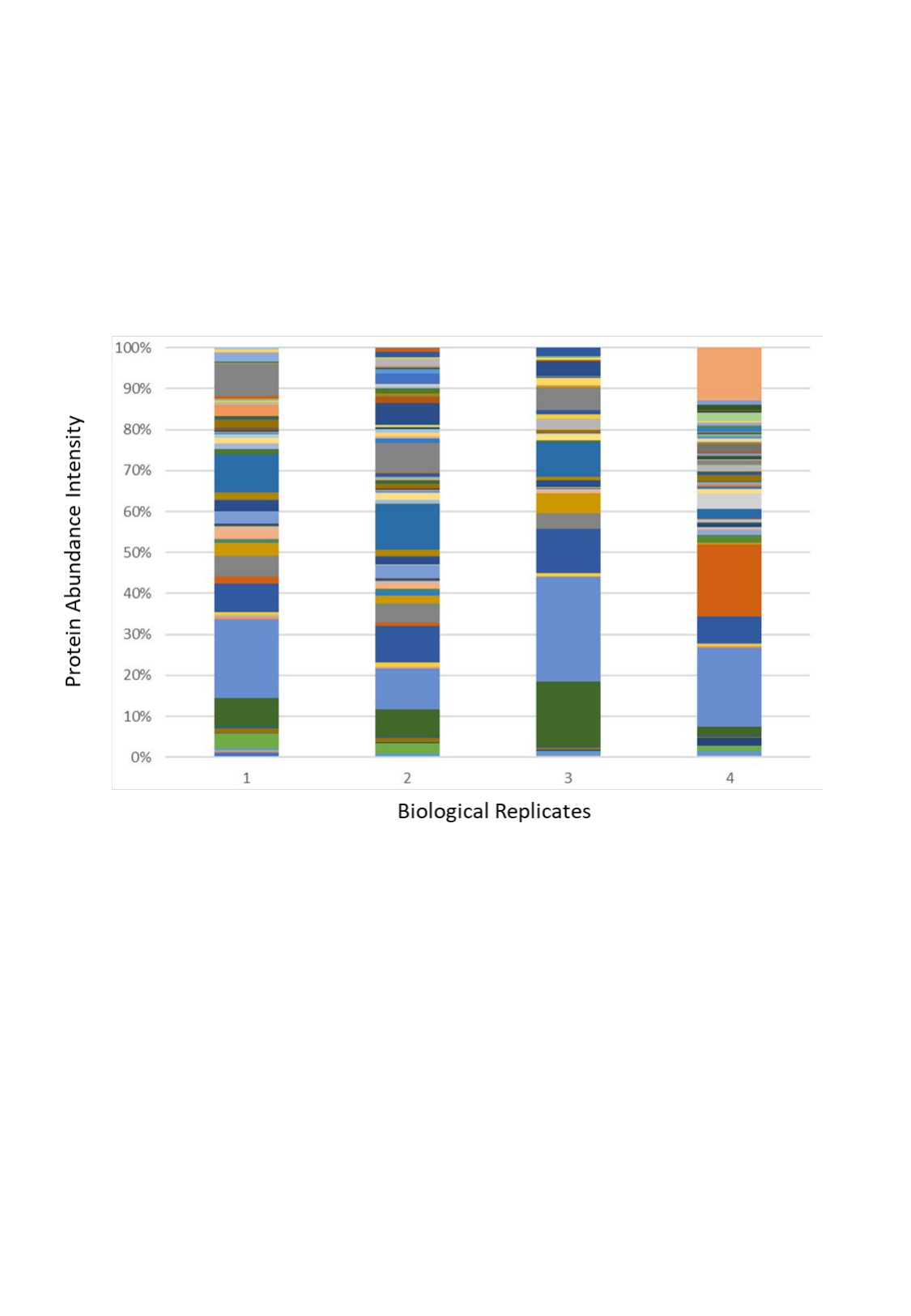

## Slide 2
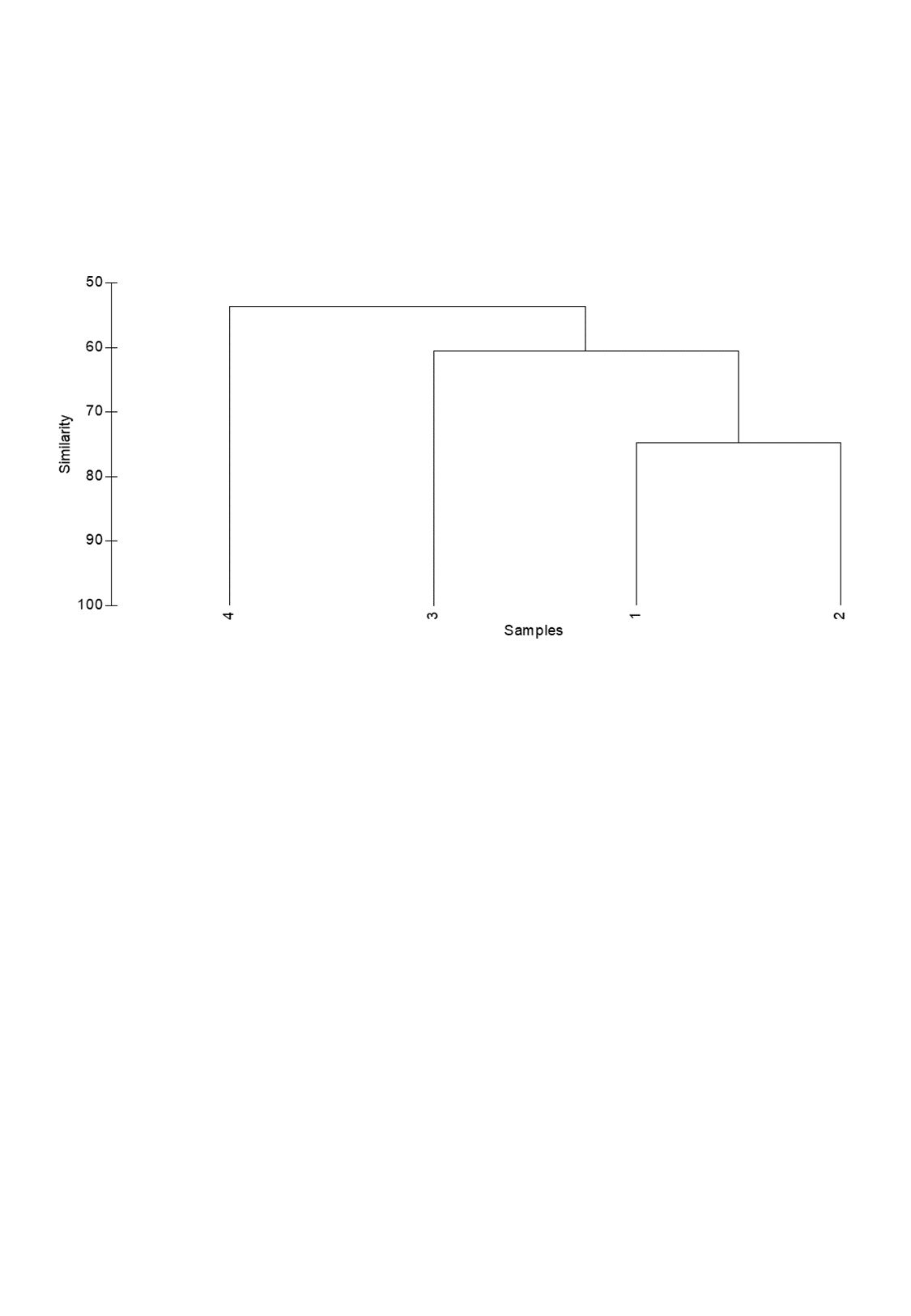

Supplement: Supplementary file 1 [file ijms-25-03739-s001.zip › Figure S3.pptx]

## Slide 1
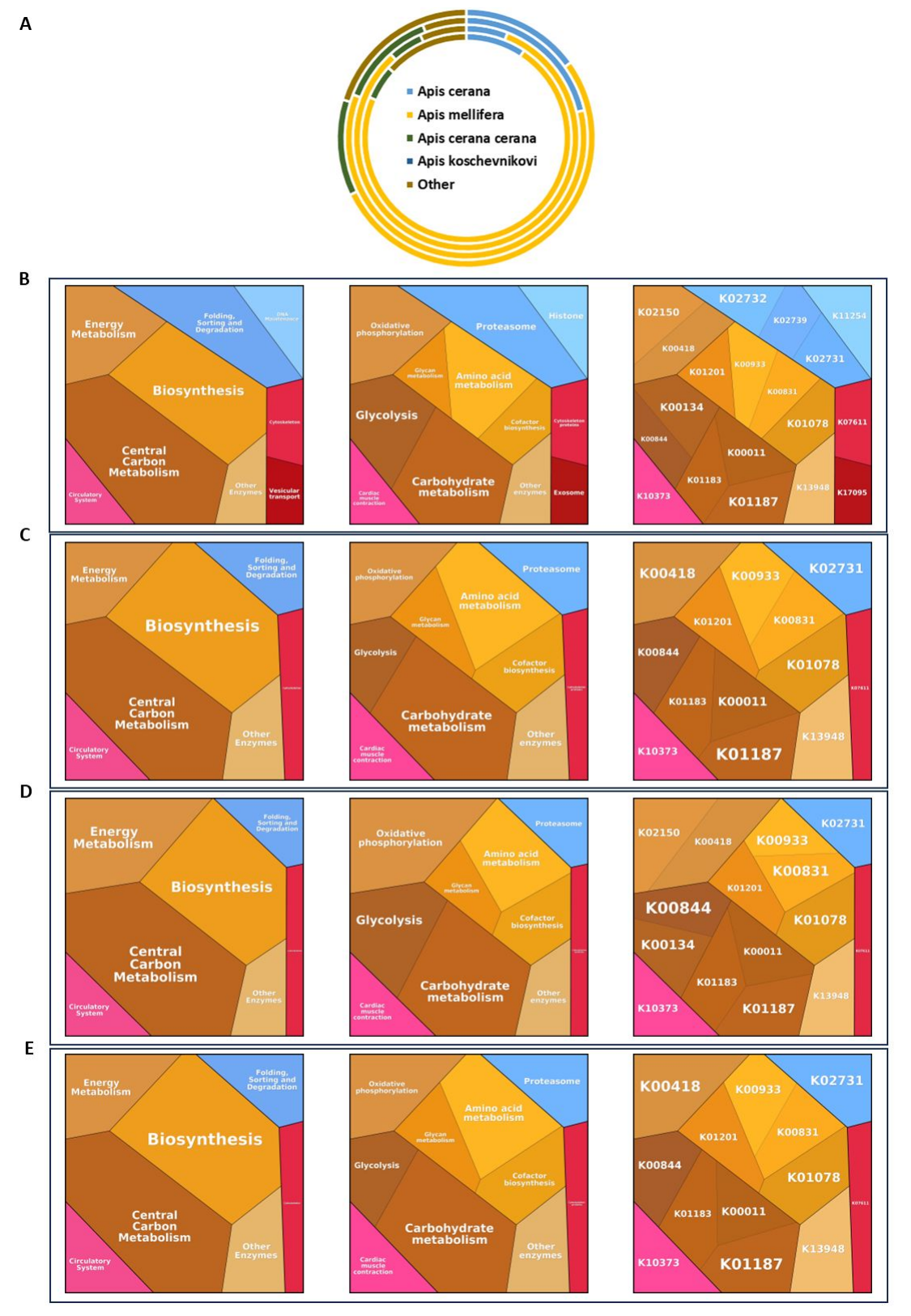

Supplement: Supplementary file 1 [file ijms-25-03739-s001.zip › Figure S4.pptx]
